# Rheumatoid Synovial Fluid and Acidic Extracellular pH Modulate the Immunomodulatory Activity of Urine-Derived Stem Cells

**DOI:** 10.3390/ijms242115856

**Published:** 2023-11-01

**Authors:** Michaela Cehakova, Dana Ivanisova, Magdalena Strecanska, Jana Plava, Zuzana Varchulova Novakova, Andreas Nicodemou, Stefan Harsanyi, Martina Culenova, Sona Bernatova, Lubos Danisovic

**Affiliations:** 1 National Institute of Rheumatic Diseases, Nabrezie I. Krasku 4, 921 12 Piestany, Slovakia; magdalena.strecanska@nurch.sk (M.S.); zuzana.varchulova@fmed.uniba.sk (Z.V.N.); andreas.nicodemou@fmed.uniba.sk (A.N.); stefan.harsanyi@fmed.uniba.sk (S.H.); martina.culenova@fmed.uniba.sk (M.C.); lubos.danisovic@fmed.uniba.sk (L.D.); 2 Institute of Medical Biology, Genetics and Clinical Genetics, Faculty of Medicine, Comenius University Bratislava, Sasinkova 4, 811 08 Bratislava, Slovakia; dana.ivanisova@fmed.uniba.sk (D.I.); jana.plava@savba.sk (J.P.); sona.bernatova@fmed.uniba.sk (S.B.); 3 Biomedical Research Center of the Slovak Academy of Sciences, Dubravska Cesta 9, 845 05 Bratislava, Slovakia

**Keywords:** rheumatoid arthritis (RA), rheumatoid synovial fluid (RASF), acidic extracellular pH, urine-derived stem cells (UdSCs), immunomodulation

## Abstract

Urine-derived stem cells (UdSCs) possess a remarkable anti-inflammatory and immune-modulating activity. However, the clinical significance of UdSCs in autoimmune inflammatory diseases such as rheumatoid arthritis (RA) is yet to be explored. Hence, we tested the UdSCs response to an articular RA microenvironment. To simulate the inflamed RA joint more authentically in vitro, we treated cells with rheumatoid synovial fluids (RASFs) collected from RA patients, serum deprivation, acidosis (pH 7.0 and 6.5), and their combinations. Firstly, the RASFs pro-inflammatory status was assessed by cytokine quantification. Then, UdSCs were exposed to the RA environmental factors for 48 h and cell proliferation, gene expression and secretion of immunomodulatory factors were evaluated. The immunosuppressive potential of pre-conditioned UdSCs was also assessed via co-cultivation with activated peripheral blood mononuclear cells (PBMCs). In all experimental conditions, UdSCs’ proliferation was not affected. Conversely, extracellular acidosis considerably impaired the viability/proliferation of adipose tissue-derived stem cells (ATSCs). In the majority of cases, exposure to RA components led to the upregulated expression of IL-6, TSG6, ICAM-1, VCAM-1, and PD-L1, all involved in immunomodulation. Upon RASFs and acidic stimulation, UdSCs secreted higher levels of immunomodulatory cytokines: IL-6, IL-8, MCP-1, RANTES, GM-CSF, and IL-4. Furthermore, RASFs and combined pretreatment with RASFs and acidosis promoted the UdSCs-mediated immunosuppression and the proliferation of activated PBMCs was significantly inhibited. Altogether, our data indicate that the RA microenvironment certainly has the capacity to enhance UdSCs’ immunomodulatory function. For potential preclinical/clinical applications, the intra-articular injection might be a reasonable approach to maximize UdSCs’ therapeutic efficiency in the RA treatment.

## 1. Introduction

Worldwide attention has been directed towards adult stem cells, particularly mesenchymal/medicinal stem/signaling cells (MSCs), owing to their extensive regenerative potential. Therefore, they are currently involved in hundreds of clinical trials [1]. MSCs have not only tissue-repairing but also immunoevasive, anti-inflammatory, and immunosuppressive properties. In fact, they show only weak expression of the major histocompatibility complex (MHC) class I molecules and lack the expression of MHC class II molecules. MSCs are able to interfere with both the adaptive and innate immune systems. The mechanisms behind immunomodulation include cell-to-cell interaction as well as secretion of bioactive agents and/or particles engaged in paracrine signaling [2,3].

MSCs are not immunomodulatory ‘by default’, they acquire this phenotype upon contact with immune cells or stimulation with pro-inflammatory cytokines, particularly interferon-γ (IFN-γ), alone or in combination with interleukin-1β (IL-1β) or tumor necrosis factor (TNF) [4,5]. Once activated, MSCs suppress T cell proliferation and function and/or shift the phenotype of T cells to that of functional regulatory T cells (Tregs); support Treg proliferation and activation; inhibit B cell proliferation, differentiation, and immunoglobulin (Ig) production; trigger macrophage polarization towards the anti-inflammatory phenotype; and block dendritic cell maturation as well as natural killer (NK) cells [3,4]. The pro-inflammatory stimulus induces the upregulation of several targets, such as intercellular adhesion molecule 1 (ICAM-1), vascular cell adhesion molecule 1 (VCAM-1), and programmed death ligand 1 (PD-L1). The first two of these molecules are fundamental for mediating the adhesion of MSCs to T cells. This intercellular interaction causes the functional inhibition of pro-inflammatory cells through a co-inhibitory signal transmitted by PD-L1 [6,7,8]. Besides direct cell-to-cell contact, MSCs also operate via secretion of immune inhibitory factors including indoleamine-1,2-dioxygenase (IDO), prostaglandin E2 (PGE2), interleukin 6 (IL-6), interleukin 10 (IL-10), transforming growth factor beta (TGF-β), and tumor necrosis factor-stimulated gene 6 (TSG6), affecting T cells, NK cells, and macrophages [3,9].

Based on in vitro as well as in vivo experiments, MSCs have displayed potent immunosuppressive and anti-inflammatory activity with which to dampen joint injuries in autoimmune inflammatory diseases, such as rheumatoid arthritis (RA) [10,11,12]. However, in human clinical trials, with predominantly intravenous application of MSCs, only a moderate response to therapy has been observed. On the other hand, a very promising outcome in terms of safety has been reported, as RA patients have experienced neither toxicity nor adverse effects [13,14]. These results show that MSCs can still be used as a therapeutic tool, but that their immunomodulatory abilities need to be improved. Appropriate cues from the pro-inflammatory microenvironment could allow MSCs to be fully activated. Arthritic joints, characterized by chronic synovitis and progressive bone and cartilage damage, are overloaded with various cytokines and chemokines e.g., TNF-α, IL-1, IL-6, IL-12, IL-17, IL-18, IL-21, GROα, and RANTES. All of these pro-inflammatory mediators might have a potential role in priming the immunomodulatory function of MSCs to attenuate inflammation and suppress the over-activated immune system [15,16].

Noteworthily, there are also other biological phenomena that play a critical role in the RA pathogenesis and might considerably affect the efficiency of not only MSCs administration but also anti-RA therapy in general. For instance, it has been known for over 50 years that rheumatoid synovial fluids (RASFs) are typically acidic [17,18,19]. Paradoxically, studies investigating the effects of acidic stress in the context of the inflammatory microenvironment are still missing.

Multiple tissues can be used to collect MSCs: they have been isolated from adipose tissue, bone marrow, umbilical cord, periosteum, synovium, and others. Urine-derived stem cells (UdSCs) represent a newly discovered type of stem cells that possess similar biological features to MSCs. What makes them even more attractive is the fact that they can be easily harvested via spontaneous urine voiding in a non-invasive as well as low-cost method [20]. Furthermore, UdSCs have been shown to have a potent anti-inflammatory and immune-modulating activity [21,22,23]. However, to our knowledge, there is no evidence demonstrating their therapeutic potential in the RA scenario. Therefore, we decided to test UdSCs response to an articular RA microenvironment.

Here, for the first time, we have analyzed how different factors of the synovial environment of RA patients (different level of inflammatory mediators, acidosis, and lack of nutrients) influence UdSCs’ biological characteristics (proliferation/viability, gene expression and secretion of immunomodulatory factors, and immunosuppression of activated peripheral blood mononuclear cells (PBMCs)) in a small pilot study. To mimic the articular RA microenvironment more authentically, we exposed UdSCs to RASFs (1 mg/mL), nutrient shortage, extracellular acidosis (pH 7.0 and 6.5), and their combinations. We found that UdSCs showed great adaptability and increased immunomodulatory activity under conditions resembling the inflamed joints of RA patients. On the contrary, acidic stress significantly impaired the viability/proliferation of adipose tissue-derived stem cells (ATSCs). It is plausible that UdSCs might survive, and even more importantly have a therapeutic effect, after intra-articular delivery to the affected RA joints. Thus, our results provide a valuable suggestion for pushing the UdSCs research forward into at least a preclinical model of RA studies.

## 2. Results

### 2.1. RASFs Characterization According to Patients’ Data and Severity of Inflammation

**Figure 1:**
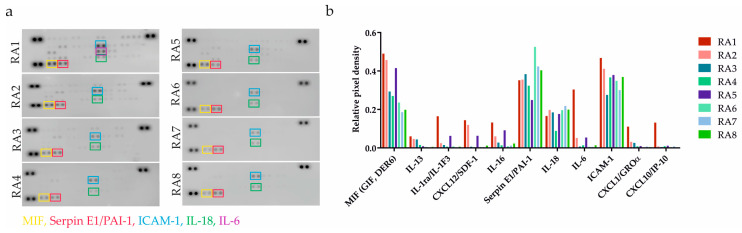
Evaluation of inflammatory status of RASFs. (**a**) Pro-inflammatory mediators were detected using an antibody-based membrane array in RASFs from 8 RA patients. Black spots on the membrane represent each cytokine spotted in duplicates. Each colorful frame refers to one cytokine. (**b**) Different severity of inflammation was confirmed based on number of detected cytokines and signal intensity. The relative change in cytokines level was determined by subtraction of each pair of capture antibody from the reference spot signal on the corresponding membrane. SFs from patient RA1 and RA2 were determined as strongly pro-inflammatory. SFs from patients RA3-8 were confirmed as less inflammatory.

RA patients’ clinical characteristics can be found in Table 1. To ascertain the pro-inflammatory status of RASFs, we performed an initial screening of 36 different cytokines by applying a cytokine-profiling array (Figure 1a). In addition to the cytokines and chemokines that are generally associated with inflamed RA joints (such as IL-1RA, IL-6 or IL-18), we also identified proteins including macrophage migration inhibitory factor (MIF), plasminogen activator inhibitor 1 (PAI-1), and soluble ICAM-1 (sICAM-1) that are not extensively discussed but are also implicated in the RA pathogenesis [24,25,26]. Paradoxically, TNF-α and IFN-γ concentrations were generally low, which is in line with the Proteome Profiler Array data published by Sayegh et al. [7].

SF from patient RA1 followed by RA2 was confirmed as strongly pro-inflammatory. Samples from patients RA3-8 were determined as less inflammatory based on quantitative evaluation (Figure 1b). The profiling analysis data led to the exclusion of patient RA4’s specimen due to the small residual volume of the sample.

### 2.2. RASF Treatment Preserved the Morphological Characteristics and Proliferative Activity of UdSCs

**Figure 2:**
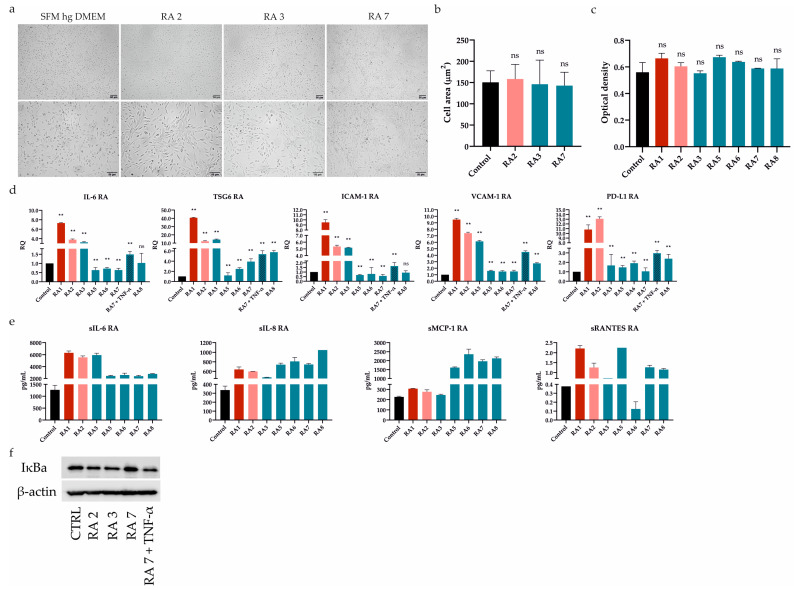
Differential effect of RASFs on UdSCs morphology, proliferation, gene expression, secretion, and protein level of IκB. UdSCs were cultured in an SFM with (treatment) or without RASFs (control, CTRL) at 1 mg/mL for 48 h. RASFs are listed depending on their inflammatory status (red being very pro-inflammatory, pink moderate, and blue less inflammatory). UdSCs were also cultured in an SFM in the presence of TNF-α (10 ng/mL) under less inflammatory conditions mediated by the RA7 SF for 48 h. (**a**) Cell cultures were analyzed under a light microscope (Zeiss Axiovert 100) using 40× (upper panel) and 100× (lower panel) magnifications. Representative pictures of UdSCs after 48 h RASF treatment with different pro-inflammatory statuses: moderate (RA2) and low (RA3 and RA7). (**b**) Quantitative analysis of the cell area demonstrated that RASF incubation did not show negative impact on the UdSCs morphological features. (**c**) Proliferative capacity was assessed using a colorimetric MTS proliferation assay. RASFs did not interfere with the proliferative activity. (**d**) IL-6, TSG6, ICAM-1, VCAM-1, and PD-L1 gene expressions were assessed using quantitative RT-PCR. Highly pro-inflammatory SF from RA1 very potently enhanced the expression of all targets in contrast with the less inflammatory RASFs. GAPDH served as an internal control. (**e**) After 48 h pre-incubation with RASFs cells were kept in an SFM for another 48 h to obtain conditioned media. Analysis of the cells’ secretory activity demonstrated that sIL-6 was secreted at the highest concentrations (ranged from 2397 to 6317 pg/mL) followed by sMCP-1 (ranged from 246 to 2361 pg/mL). Moderate levels were measured for sIL-8 (ranged from 475 to 1048 pg/mL). The lowest concentrations within the conditioned media were identified for sRANTES (0.1–2.2 pg/mL). (**f**) Western blot analysis of IĸB protein expression showed that NFkB signaling might be involved in the regulation of UdSCs-mediated immunomodulation. Very high IκB levels, indicating inhibition of NFkB, were associated with the less inflammatory SF from RA7, which had the overall weakest effect on the expression of immunomodulatory genes in UdSCs. Results are presented as mean ± SD. ** *p* < 0.01. ns, non-significant.

First of all, we tested the effect of seven RASFs on UdSCs’ behavior. Cells were cultured in a commercial serum-free (SFM) high-glucose (hg) DMEM (SFM hg DMEM) with (treatment) or without RASFs (control, CTRL) at 1 mg/mL concentration for 48 h. We did not observe any morphological changes as a consequence of RASF incubation (Figure 2a), which was confirmed by the cell area measurement (Figure 2b). Likewise, all RASFs maintained the UdSCs proliferative activity, and differences were found to be insignificant (Figure 2c).

### 2.3. RASFs Differentially Modulated Gene Expression and Secretion of Immunomodulatory Factors in UdSCs

It has been previously shown that ATSCs-mediated immune regulation can be improved by RASF pretreatment. This pro-inflammatory stimulus resulted in the upregulated expression of immunomodulatory (IL-6, TSG6) and immune suppressive (ICAM-1, VCAM-1, and PD-L1) genes [7]. As these mechanisms are also expected to operate in UdSCs, we performed RT-PCR analysis after a 48 h pre-incubation with RASFs (1 mg/mL). To further confirm the impact of inflammation, we cultured UdSCs in the presence of the RA-associated pro-inflammatory cytokine TNF-α (10 ng/mL) under less inflammatory conditions mediated by the RA7 SF for 48 h (Figure 2d). Highly pro-inflammatory SF from patient RA1 strongly induced the expression of all genes (up to a 40-fold increase) compared with UdSCs cultured in an SFM without RASF (control, CTRL). RA2 SF can be considered as moderate, which was mirrored by the potent stimulation of gene expression (up to a 13-fold induction) in comparison with untreated control. On the other hand, in general, less inflammatory RASFs had more modest effects on the expression induction of all studied genes. Strikingly, the downregulated expression of genes encoding IL-6 and ICAM-1 was detected as a consequence of RA5-7 SFs pre-incubation. A combination of TNF-α and less inflammatory RA7 SF resulted in the upregulation of all studied genes. However, TNF-α did not induce the expression as much as highly pro-inflammatory RA1 SF. Thus, we suppose that, besides TNF-α, there are also other pro-inflammatory mediators operating within the RA environment that are involved in the enhancement of UdSCs’ immune-modulating potential (Figure 2d).

To further examine the immunomodulatory ability of UdSCs, we analyzed their secretome after exposure to RASFs (1 mg/mL). As a control, UdSCs kept in an SFM without RASF were used. Generally, treatment with RASFs led to increased secretion of all analyzed soluble molecules (sIL-6, sIL-8, sMCP-1, sRANTES), albeit to different extents (Figure 2e). sRANTES represented the only exception as its secretion decreased after RA6 SF treatment. Our results further reveal that sIL-6 exhibited the same profile in secretion as in gene expression (Figure 2d vs. Figure 2e). In the case of sIL-8 and sMCP-1, pro-inflammatory RASFs (RA1, RA2) showed weaker effects on their secretion than low-inflammatory RA5-8 SFs. The secretion of sRANTES did not align with the inflammatory status of RASFs in any aspect.

Upon exposure to RASFs, NFkB was confirmed to be crucial for the enhancement of ATSCs’ immunomodulatory potential [7]. Therefore, we investigated whether UdSCs stimulated with RASFs with different pro-inflammatory statuses displayed different expressions of IκB, which is inversely correlated with NFkB activation. The less inflammatory SF from RA7, with the overall weakest effect on gene expression, as depicted in Figure 2d, showed very high IκB levels compared with other RASFs, indicating inhibition of NFkB. Interestingly, RA3 SF, even with a low pro-inflammatory profile, exhibited IκB protein expression comparable with moderate RA2 SF—this is in line with their effects on gene expression, except PD-L1. To verify if the NFkB pathway is active in UdSCs, we cultured cells in an SFM hg DMEM with RA7 SF in combination with TNF-α (10 ng/mL) (RA7 + TNF-α). TNF-mediated activation of NFkB signaling was confirmed by a decreased level of IκB (Figure 2f). Our findings suggest that the NFkB pathway can be partially implicated in the inflammation-associated amelioration of UdSCs immunomodulatory potential.

### 2.4. Acidic Extracellular pH Did Not Affect UdSCs’ Proliferative Activity but Impaired ATSCs’

**Figure 3:**
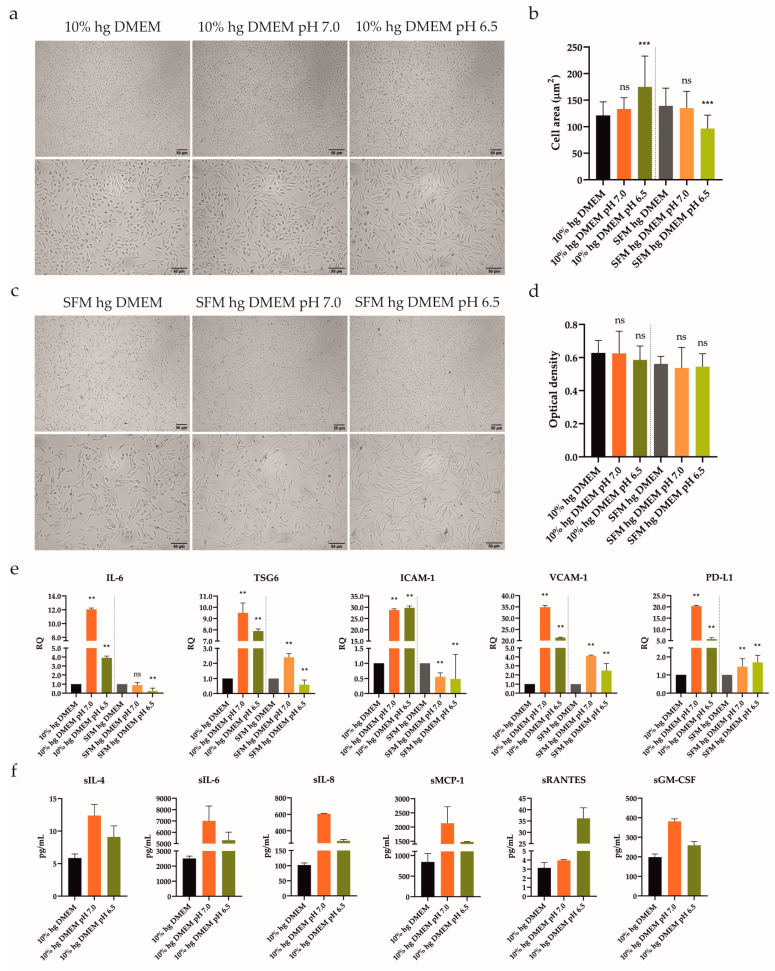
Influence of acidic stress (pH 7.0 and 6.5) with or without 10% FBS on UdSCs morphological features, proliferative capacity, gene expression, and secretion. Cells were analyzed after 48 h exposure to acidic media with or without 10% FBS. (**a**,**c**) Cells were subjected to light microscope analysis (Zeiss Axiovert 100) using 40× (upper panel) and 100× (lower panel) magnifications. (**b**) Quantitative analysis of cell area confirmed the impact of acidic stimulation on cell morphology. Serum-free conditions combined with acidosis (pH 6.5) had the most significant effect on cell shape. (**d**) Proliferative capacity was investigated using a colorimetric MTS proliferation assay. Acidic stimulation maintained proliferative activity with, as well as without, 10% FBS. (**e**) IL-6, TSG6, ICAM-1, VCAM-1, and PD-L1 gene expressions were evaluated using quantitative RT-PCR. Mild acidosis (pH 7.0), regardless of FBS, potently stimulated the expression of studied genes, besides ICAM-1. GAPDH served as an internal control. (**f**) Cytokine profile of conditioned media from cells pre-incubated with an acidic pH (7.0 and 6.5) in the presence of 10% FBS. After 48 h pre-treatment, cells were cultured in an SFM for another 48 h to obtain conditioned media. Analysis of the cells’ secretory activity uncovered that pH 7.0 had a stronger effect on the secretion rate of all studied proteins compared with pH 6.5, besides sRANTES. sIL-6 was secreted at the highest concentrations (7007 vs. 5309 pg/mL) followed by sMCP-1 (2135 vs. 1474 pg/mL). Moderate levels were measured for sIL-8 (607 vs. 278 pg/mL) and sGM-CSF (381 vs. 259 pg/mL). The lowest concentrations within conditioned media were detected for sRANTES (4 vs. 36 pg/mL) and sIL-4 (12 vs. 9 pg/mL). Results are presented as mean ± SD. ** *p* < 0.01; *** *p* < 0.001. ns, non-significant.

The synovial RA microenvironment is characterized by persistent inflammation, which has been scrutinized for over a decade [14]. Despite the fact that inflamed joints are also related to local acidosis, those effects have not been studied extensively. Thus, we evaluated the alterations in morphology and viability/proliferation of UdSCs after acidic treatment. Cells were cultured in media with an acidic pH (pH 7.0 and 6.5) with or without 10% FBS for 48 h, and then assayed. Cells cultured in a commercial hg DMEM with or without 10% FBS were used as controls. UdSCs showed a change in cell shape after being exposed to acidic stimulation with pH 6.5, in the presence of 10% FBS (Figure 3a)—a morphological alteration was confirmed by the measurement of the cell area (Figure 3b). Severe serum-free conditions (without FBS) in the combination with acidosis (pH 6.5) had an even more pronounced impact on cell morphology (Figure 3b). Cells acquired an elongated shape compared with control UdSCs (Figure 3c). However, the cell viability and MTS test indicated that acidic stress (with or without FBS) did not negatively interfere with cell viability or proliferation (Figure 3d).

**Figure 4:**
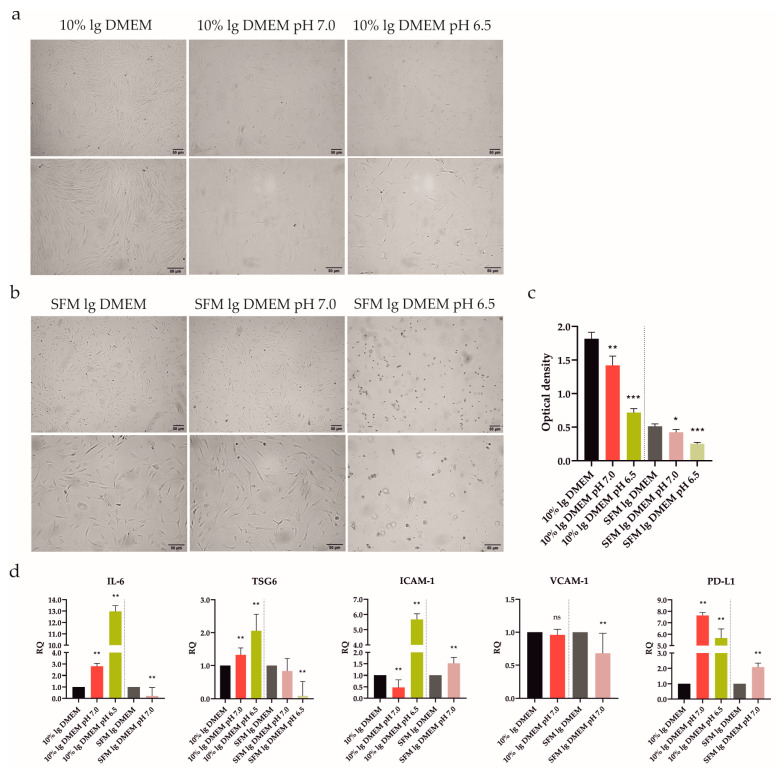
Impact of extracellular acidic stimulation (pH 7.0 and 6.5) with or without 10% FBS on ATSCs’ morphological characteristics, proliferation, and gene expression after 48 h incubation. (**a**,**b**) Cells were analyzed under a light microscope (Zeiss Axiovert 100) using 40× (upper panel) and 100× (lower panel) magnifications. After 48 h exposure to acidic conditions combined with or without 10% FBS cells exhibited an elongated shape. (**b**) Extracellular acidosis had a negative effect on ATSCs, the shortage of nutrients in combination with acidic stress (pH 6.5) even inducing ATSCs to apoptosis. (**c**) Proliferative activity was determined using a colorimetric MTS proliferation test. Acidic stimulation significantly decreased the cell proliferation with, as well as without, 10% FBS. (**d**) IL-6, TSG6, ICAM-1, VCAM-1, and PD-L1 gene expressions were determined using quantitative RT-PCR. In the presence of 10% FBS, both acidic media upregulated IL-6, TSG6, PD-L1, and pH 6.5 was even able to upregulate ICAM-1. A nutrient deprivation combined with harsh acidic stress (pH 6.5) induced cell death, as no signal was detected, except for a very low one for TSG6. GAPDH served as an internal control. Results are presented as mean ± SD. * *p* < 0.05; ** *p* < 0.01; *** *p* < 0.001.

ATSCs have shown potent immune-modulating properties after treatment with SFs from RA patients [7]. However, after intra-articular delivery, they will encounter the severe conditions of the inflamed joint, including acidosis. ATSCs’ behavior in an acidic environment has not yet been explored. Consequently, we elucidated whether acidic stress (pH 7.0 and 6.5) could modulate the ATSCs morphology and viability/proliferation. The experiment was set up precisely in the same manner as described above with UdSCs, with one exception: instead of hg, the low glucose (lg) DMEM was used. Similar to UdSCs, ATSCs exhibited elongated morphology as a result of acidic stimulation with as well as without 10% FBS. Nonetheless, it is clear that extracellular acidosis significantly harmed ATSCs (Figure 4a,b). Harsh acidosis (pH 6.5) had the most devastating effect on ATSCs’ viability (Table 2). The ATSCs’ proliferative activity was inhibited considerably by both acidic conditions compared with controls (10% FBS lg and SFM lg DMEM), as shown in Figure 4c. Overall, UdSCs appear to be more adaptable to acidic stress compared with ATSCs.

### 2.5. Acidic Stress Diversely Influenced UdSCs’ and ATSCs’ Immunomodulatory Potential

To obtain a deeper insight into the changes on the mRNA level, we investigated the expression of genes linked to immunomodulatory activity (IL-6, TSG6, ICAM-1, VCAM-1, and PD-L1). UdSCs and ATSCs were cultured in media with an acidic pH (7.0 and 6.5), with or without 10% FBS. After 48 h our data revealed that, in UdSCs, mild acidosis (pH 7.0) with 10% FBS strongly induced the expression of all genes (up to a 35-fold increase) compared with harsh acidosis (pH 6.5), with the exception of ICAM-1. This trend was also maintained in nutrient-deprived conditions but with overall weaker induction, as shown in Figure 3e. Notably, lack of nutrients, in combination with mild acidosis (pH 7.0), inhibited the expression of ICAM-1 (Figure 3e). When combined with harsh acidic stress (pH 6.5), the expressions of IL-6, TSG6, and ICAM-1 were downregulated compared with the control (Figure 3e).

ATSCs’ proliferation was significantly diminished as a result of pre-incubation with an acidic pH. Therefore, as a next step, we verified the way in which this effect could interfere with the regulation on the mRNA level (Figure 4d). Interestingly, ATSCs were able to respond by upregulated expression of IL-6, TSG6, ICAM-1, and PD-L1 after exposure to severe acidosis (pH 6.5) in the presence of 10% FBS. Mild acidosis (pH 7.0) in combination with 10% FBS increased the expression of IL-6, TSG6, and PD-L1, whilst the expression of ICAM-1 decreased. In line with the MTS data (Figure 4c), a shortage of nutrients combined with harsh acidic stress (pH 6.5) triggered cell death, as we could not detect any signal except for a very low one for TSG6. Serum-free conditions in combination with mild acidosis (pH 7.0) significantly upregulated ICAM-1 and PD-L1 and downregulated IL-6 and VCAM-1 compared to the control.

Based on the RT-PCR analysis, we found that UdSCs were more sensitive to acidic stimulation, which enhanced the expression of studied genes very potently, especially in the presence of 10% FBS (up to a 35-fold induction) compared with the control (Figure 3e). On the other hand, ATSCs seemed to be less responsive (up to a 13-fold increase) (Figure 4d). Furthermore, ATSCs’ proliferative capacity was significantly diminished under both acidic conditions, with and without 10% FBS (Figure 4c). Hence, we proceeded with further experiments using UdSCs only. To assess their extended secretion profile, an analysis of conditioned media was performed in cells exposed to acidic stimulation with 10% FBS. As a control, UdSCs cultured in 10% FBS commercial hg DMEM were used. After 48 h the cells were washed twice with PBS and cultured in an SFM for another 48 h. Acidic stress elevated the secretion of all studied soluble targets (sIL-4, sIL-6, sIL-8, sMCP-1, sRANTES and sGM-CSF) (Figure 3f). However, the influence of mild acidosis (pH 7.0) on the secretion rate was more pronounced (except for sRANTES) compared with pH 6.5.

### 2.6. Adding Pro-Inflammatory Mediators into Acidic Conditions Blocked Neither UdSCs’ Proliferation nor Transcription of Immunomodulatory Genes

**Figure 5:**
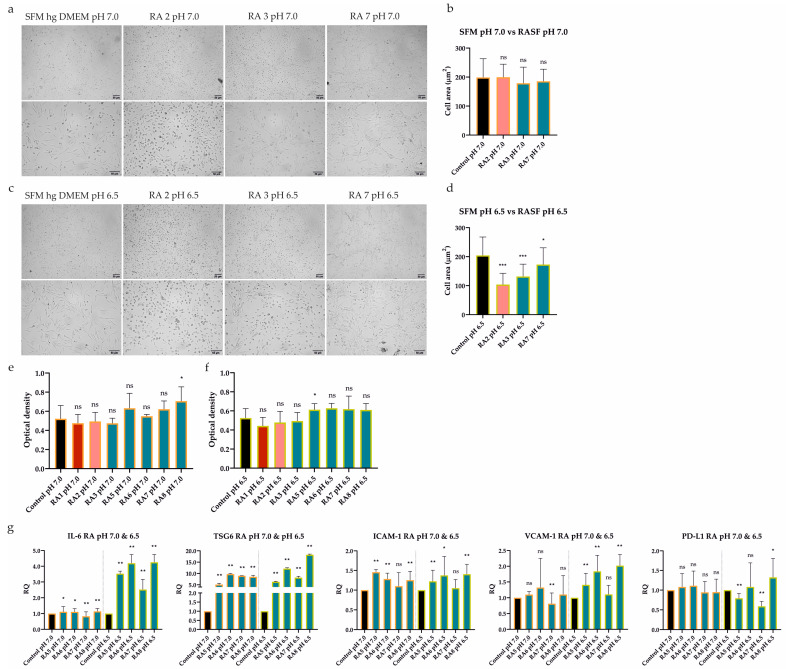
Effect of extracellular acidosis (pH 7.0 and 6.5) combined with inflammation on UdSCs’ morphological characteristics, proliferative capacity, and gene expression. Cells were cultured in an acidic SFM hg DMEM media with (treatment) or without RASFs (control) at 1 mg/mL for 48 h. RASFs are listed depending on their inflammatory status (red being very pro-inflammatory, pink moderate and blue less inflammatory). (**a**,**c**) Cells were analyzed under a light microscope (Zeiss Axiovert 100) using 40× (upper panel) and 100× (lower panel) magnifications. (**a**,**b**) No significant impact on cell morphology under mild acidosis (pH 7.0) and moderate or low inflammatory stimuli was detected upon quantitative measurement of cell area. (**c**,**d**) The inflammatory environment was related to the alteration in cell shape under harsh acidosis (pH 6.5). Moderate RA2 SF had the most profound effect on cell morphology, which was confirmed by the cell area measurement. (**e**,**f**) Cell proliferation was measured by a colorimetric MTS proliferation test. Acidic stimulation in combination with different levels of inflammation did not significantly suppress the proliferative activity in any treatment variant. (**g**) IL-6, TSG6, ICAM-1, VCAM-1, and PD-L1 gene expression was evaluated by quantitative RT-PCR. Generally, exposure to acidic stress combined with low-severity inflammation elevated the expression of analyzed genes, despite harsh acidosis (pH 6.5) seeming to have had a stronger effect. GAPDH served as an internal control. Results are presented as mean ± SD. * *p* < 0.05; ** *p* < 0.01; *** *p* < 0.001.

Having shown that the immunomodulatory activity of UdSCs can be regulated by pro-inflammatory mediators and acidic stress separately, we further examined the effect of their combination. Cells were cultured in an acidic SFM hg DMEM with (treatment) or without RASFs (control) at 1 mg/mL for 48 h. Then, the morphology, proliferative activity, and expression of genes implicated in immunomodulation were analyzed. The addition of moderate (RA2) and less inflammatory (RA3, RA7) SFs had no significant impact on cell morphology under mild acidosis (Figure 5a,b). On the contrary, inflammatory stimuli were associated with a change in cell shape under harsh acidosis (pH 6.5), with cells being the most affected upon RA2 SF treatment (Figure 5c,d). An MTS test, however, demonstrated that, under inflammatory conditions, the proliferation was inhibited neither in mild acidic stress (Figure 5e) nor severe acidosis (Figure 5f). Of important note, UdSCs were able to survive the severe conditions of nutrient deprivation, acidosis, and inflammation without any significant damage, pointing to their great plasticity.

Further, the gene expression of immunomodulatory factors was assessed. We tested whether UdSCs treated with less inflammatory RASFs (RA5-8) combined with acidic stress (pH 7.0 and pH 6.5) for 48 h would be still able to execute the immunomodulatory activity. Our evidence indicates that, in the majority of cases, the exposure to acidic stress combined with low-severity inflammation resulted in upregulated expression, albeit to different extents (Figure 5g). Under the inflammatory stimulus, TSG6 can be considered the most responsive both in mild (up to a 9-fold induction) and harsh acidosis (up to an 18-fold increase). On the other hand, PD-L1 showed the weakest induction as elevated expression was detected only in the presence of RA8 SF in severe acidosis.

### 2.7. RASFs and Combined RASFs + Acidosis Pre-Conditioning Augments the Inhibitory Effect of UdSCs on PBMCs Proliferation

**Figure 6:**
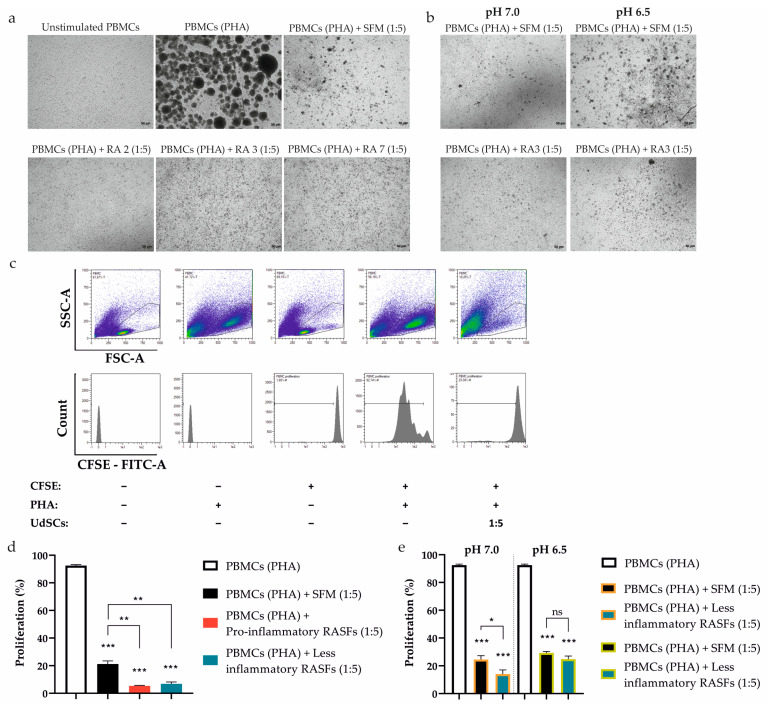
Effect of UdSCs pre-conditioned with RASFs and RASFs within an acidic background (pH 7.0 and pH 6.5) on the proliferation of CFSE-labelled and PHA-activated peripheral blood mononuclear cells (PBMCs (PHA)). UdSCs were cultured in a commercial SFM hg DMEM with (treatment) or without RASFs (control) at 1 mg/mL or in an acidic SFM hg DMEM media with (treatment) or without RASFs (control) at 1 mg/mL. After 48 h, UdSCs were washed twice with PBS and directly co-cultured with carboxyfluorescein succinimidyl ester (CFSE)-labelled PBMCs stimulated with PHA (10 µg/mL) (PBMCs (PHA)) at a cell ratio of 1:5 for another 4 days. (**a**,**b**) Cells were analyzed under a light microscope (Zeiss Axiovert 100) using 40× magnification. Representative pictures of RASFs- and RASFs + acidosis-pretreated UdSCs after 4 day co-cultivation with PBMCs (PHA). (**c**) Representative flow cytometry images showing the influence of co-culture of PBMCs (PHA) and UdSCs on the proliferative capacity of PBMCs (PHA). The top panels of density plots (showing the distribution of events from low to high by blue, green, and red color, respectively) represent the gating strategy by which to target the single cells within the PBMC population, while the lower panel displays the fluorescence intensity of CFSE on the gated PBMCs. Proliferation was determined by measurement of CFSE dilution (displayed as percent gated (%-#). (**d**) The effect of the co-culture of PBMCs (PHA) and RASF-pre-incubated UdSCs on PBMC (PHA) proliferation. RASFs are listed depending on their inflammatory status (red being pro-inflammatory and blue less inflammatory). RASF-pre-incubated UdSCs, regardless of the pro-inflammatory profile, exhibited strong immunosuppressive ability. They significantly inhibited the PBMCs’ proliferation compared with the proliferative capacity of PBMCs co-cultured with control UdSCs without RASF pre-incubation (PBMCs (PHA) + SFM). (**e**) Impact of co-culture of PBMCs (PHA) and low inflammatory RASFs + acidosis-pre-conditioned UdSCs on PBMC (PHA) proliferation. The less inflammatory background combined with severe acidosis (pH 6.5) did not enhance the immune-modulating potential of pretreated UdSCs. Results are presented as mean ± SD. * *p* < 0.05; ** *p* < 0.01; *** *p* < 0.001. ns, non-significant.

To determine whether the pro-inflammatory stimuli- or combined inflammatory + acidic stress-pretreated UdSCs also possess an immunomodulatory activity at the cellular level, we tested their ability to attenuate the proliferation of activated PBMCs. UdSCs were cultured in a commercial SFM hg DMEM with (treatment) or without RASFs (control) at 1 mg/mL concentration for 48 h or in an acidic SFM hg DMEM media with (treatment) or without RASFs (control) at 1 mg/mL for 48 h. Then, the cells were washed twice with PBS and co-cultured with PBMCs activated with non-specific mitogenic stimuli (phytohaemagglutinin (PHA) at 10 µg/mL) for 4 days. As a control, PHA-stimulated PBMCs in the absence of UdSCs were used (PBMCs (PHA)). The monitoring of the direct UdSCs co-culture with PHA-activated PBMCs (at cell ratio of 1:5) showed that the presence of UdSCs had a strong inhibitory effect on the PBMCs’ proliferation, which was demonstrated by the reduced size and number of PBMC aggregates (Figure 6a,b). Flow cytometry data show the impact of UdSCs on the proliferation of PHA-stimulated PBMCs, as well (Figure 6c). RASF-pretreated UdSCs, regardless of the pro-inflammatory profile (pro-inflammatory RASFs vs. less inflammatory RASFs), exhibited a potent immunosuppressive ability. They significantly diminished the PBMCs’ proliferation compared with the proliferative capacity of PBMCs co-cultured with control UdSCs without RASF pre-incubation (PBMCs (PHA) + SFM), as depicted in Figure 6d. The low-level inflammation combined with mild acidosis (pH 7.0) considerably enhanced the UdSC-mediated immunosuppression compared with UdSCs pretreated in the control mild acidic condition without RASFs (PBMCs (PHA) + less inflammatory RASFs vs. PBMCs (PHA) + SFM, Figure 6e left). The less inflammatory environment combined with severe acidosis (pH 6.5), however, did not boost the immune-modulating capacity of pre-conditioned UdSCs compared with UdSCs pre-incubated in the control severe acidic condition without RASFs (PBMCs (PHA) + less inflammatory RASFs vs. PBMCs (PHA) + SFM, Figure 6e right). Taken together, we have demonstrated that UdSCs possess a remarkable immunomodulatory activity. Moreover, they have clearly shown the potential to preserve this immunosuppressive function after being exposed to an RA-related acidic environment enriched with inflammatory mediators.

## 3. Discussion

The lack of appropriate cues from inflamed RA joints might drastically decrease the efficiency of MSC-based therapy [13,14]. Indeed, direct intra-articular cell application significantly reduced the severity of antigen-induced arthritis in mice [11]. This therapeutic effect is presumably owed to various cellular mechanisms as the local RA microenvironment provides a broad scale of signals, such as pro-inflammatory mediators, acidic substances, oxidative stress, and others [14]. The pro-inflammatory stimulus is of critical importance in improving the ATSCs’ immunomodulatory activity [7]. RASFs have also been shown to promote the chondrogenic differentiation of dental follicle stem cells [27]. Moreover, acidic pre-conditioning with pH 6.8 enhanced the stem cell properties of bone marrow-derived MSCs (BM-MSCs) [28]. Nonetheless, our small study is the first to show the impact of several factors (pro-inflammatory mediators, acidosis, and serum deprivation), operating within inflamed RA joints, separately as well as in combinations. We have shown the remarkable adaptability of UdSCs not only to each factor individually but also to the harsh conditions created by their synergetic action. Our results further confirm the ability of UdSCs to acquire a different phenotype depending on the severity of the RA inflammation. Cells were more responsive to the highly/moderately pro-inflammatory SFs from patients RA1 and RA2 that potently stimulated the expression of all of the genes engaged in the immunomodulatory potential (IL-6, TSG6, ICAM-1, VCAM-1, and PD-L1). More specifically, IL-6 plays a fundamental role in mediating the effects on proliferation, phenotype regulation, and function of T cells and macrophages, whereas TSG6 promotes the shift of macrophages towards an anti-inflammatory phenotype. Furthermore, ICAM-1, VCAM-1, and PD-L1, which were strongly induced as well, are all implicated in the functional inhibition of T cells [7,9,29,30,31]. On the other hand, the less pro-inflammatory RA6 and RA7 SFs attenuated the transcription of IL-6 and ICAM-1. UdSCs’ immunomodulatory potential, however, was not completely abolished as TSG6, VCAM-1, and PD-L1 were still upregulated. Regarding cell signaling, we assume that NFκB is partially involved in the regulation of the UdSCs’ immunomodulatory potential as NFκB activity aligned with the effects of RASFs on the expression of the genes mentioned above. It is feasible that this pathway plays a non-negligible role in UdSCs-mediated immunomodulation since it has been previously reported to enhance the immunosuppressive function in ATSCs and BM-MSCs [7,32].

MSCs-mediated immunomodulation is executed by direct intercellular contact, paracrine activity, and most likely by a combination of both mechanisms [3]. Our findings demonstrate that pre-incubation with RASFs elevates the secretion of IL6, IL8, MCP-1, and RANTES in UdSCs. A higher secretion rate of IL-8, with an angiogenesis-promoting function, could be physiologically relevant, especially in the inflamed RA joints, where the vascular architecture is altered and its function can be compromised [33]. In addition, MCP-1 favors the transition of macrophages towards the anti-inflammatory phenotype [34]. This chemokine, together with RANTES, supposedly plays a role in the inhibition of immune cell proliferation. This is in agreement with previous observations published by Wu et al. The authors reported upregulated secretion of IL-6, IL-8, MCP-1, RANTES, GROα, and GM-CSF in UdSCs upon a co-culture with PBMCs. This leads to the inhibition of the PBMCs’ proliferation, underlining the biological relevance of released immunomodulatory molecules [23]. To activate UdSCs, we exposed cells to RASFs and RASFs combined with acidic stress (pH 7.0 and pH 6.5, respectively). Our data from the co-culture experiment revealed that pre-conditioned UdSCs showed an enhanced immunosuppressive activity, highlighting their medicinal potential in local RA treatment.

Maintaining the cell proliferative activity is significant for maximizing the therapeutic effect in the joint environment. UdSCs proliferated solidly in every single experimental setup (inflammatory, acidic, serum-free, and combined conditions). Oppositely, ATSCs were not capable of facing the acidic stress without significant suppression of viability/proliferation. In fact, acidosis is a common sign of the RA microenvironment. Here, the hydration of CO_2_ to proton and HCO_3_^−^ presumably represents the main pH buffering system. Therefore, to simulate the RA-related acidosis more accurately, we used bicarbonate, rather than HEPES and HCl, to adjust the pH of the culture media. The pH of SFs from patients with a variety of joint diseases (9 out of 55 with RA) ranged between 6.6 and 7.41. However, the increased SF volume creates a highly viscous environment, where microdomains with even lower pH can locally form. To test the UdSCs’ and ATSCs’ immunomodulatory potential upon acidic stimulation, we designed two models of acidosis, one with pH 7.0 (mild) and the other with pH 6.5 (harsh). Overall, UdSCs were more responsive to acidic stimulation, also under the serum-free conditions. On the cell secretion level, acidic stress increased the release of all studied molecules (sIL-6, sIL-8, sMCP-1, sRANTES, sGM-CSF, and sIL-4). Upregulated IL-4 secretion is particularly important as it operates as an anti-inflammatory cytokine. In addition, IL-4 has been demonstrated to alleviate RA symptoms by preventing joint damage and bone erosion as well as supporting tissue repair in collagen-induced arthritis mice models [35,36]. Of important note, the UdSCs’ immunomodulatory function was preserved in the presence of acidosis combined with low-level inflammation. However, the PD-L1 expression seems to be affected. On the other hand, TSG6 showed intense induction after exposure to acidic media and this effect was more pronounced in the inflammatory background.

Several limitations of this study need to be addressed in the future. First, only seven RA patients were included, and we did not analyze SF from non-rheumatologic joints. Furthermore, we worked with only four less inflammatory RASFs to investigate the combined effect of inflammation and acidosis.

Nevertheless, we have provided the first evidence demonstrating the great adaptability of UdSCs to different RA-associated environmental factors, such as inflammation, acidosis, and nutrient shortage. We have further shown that UdSCs are endowed with a potent immune-modulating function, which can be pushed forward after exposure to conditions resembling an inflamed RA joint. Despite this study being a relatively small one, our data offer an important cornerstone for future UdSCs research, potentially leading to their local preclinical/clinical application in RA therapy.

## 4. Materials and Methods

### 4.1. RASFs Collection

RASFs were obtained from 8 RA patients (7 women, 1 man). Mean patient age was 62.4 years (range: 24–82 years). Informed written consent was obtained prior to every joint aspiration and the study protocol was approved by the ethics committee of the National Institute of Rheumatic Diseases (MSC, NURCH-02-2018). All patients fulfilled the criteria for the ACR/EULAR 2010, suffered from active joint inflammation and were under the care of the National Institute of Rheumatic Diseases. RASFs were collected during the scheduled therapeutic arthrocentesis. They were transferred to heparin-treated tubes, centrifuged at 500× *g* for 10 min, and filtered on a pore size of 0.2 μm. Samples were then frozen in aliquots at −20 °C for later use.

### 4.2. RASF Characterization via Cytokine Quantification

Concentration of total RASF proteins was determined using the BCA protein assay kit (ThermoFisher Scientific, Waltham, MA, USA) according to manufacturer’s instructions. Absorbance was measured at the wavelength of 562 nm by Synergy multi-detection plate system HTX (BioTek Instruments Inc., Winooski, VT, USA). All samples for cytokine quantification were normalized to a total protein concentration of 400 μg for each RASF. Pro-inflammatory mediators were determined on all 8 RASFs using membrane-based Proteome Profiler Human Cytokine Array kit (R&D Systems, Minneapolis, MN, USA) as per the manufacturer’s instructions. The chemiluminescent signals were detected with Li-Cor Odyssey Fc Imaging System (Li-Cor Corporation, Lincoln, NE, USA) and quantitatively evaluated using ImageJ software v1.48 (NIH, Bethesda, MD, USA).

### 4.3. UdSCs Isolation and Cultivation

Urine samples were obtained from 4 healthy adults (2 men, 2 women) with no history of any severe systematic disease. The age range of the donors was 27–35 years. All donors provided informed consent and the study protocol was approved by the ethics committee of the University Hospital in Bratislava (06/2022). Spontaneously voided urine was collected, cells were isolated, cultured, and UdSCs immunophenotype and differentiation potential were characterized as previously described [21,37]. UdSCs in the earlier stages of passages (Passages 3–5) were subjected to further analysis.

### 4.4. ATSCs Isolation and Cultivation

ATSCs were obtained from 2 adult healthy individuals (1 man, 1 woman) undergoing elective tumescent abdominal liposuction. Both donors provided informed consent and all procedures were approved by the University Hospital in Bratislava (TRUSK-003). ATSCs were isolated, cultured, and their immunophenotype and differentiation potential were evaluated as stated before [38]. ATSCs in the earlier stages of passages (Passages 3–5) were further analyzed.

### 4.5. UdSCs Treatment Protocols

To investigate the effects of RASFs, extracellular acidosis (pH 7.0 and 6.5), and their combination, UdSCs were seeded in 96-well plates or 24-well plates and allowed to adhere overnight. The following day, the proliferation medium was aspirated, and cells were washed twice with PBS (Sigma-Aldrich, St. Louis, MO, USA). Control cells were cultured in commercial high-glucose DMEM (hg DMEM, Sigma-Aldrich, St. Louis, MO, USA) supplemented with 10% FBS (PAN-Biotech, Aidenbach, Germany) and Pen/Strept (Sigma-Aldrich, St. Louis, MO, USA) for 48 h. UdSCs were also cultured in commercial serum-free (SFM) hg DMEM (Sigma-Aldrich, St. Louis, MO, USA) with or without RASFs at 1 mg/mL concentration for 48 h.

DMEM (D5030, Sigma-Aldrich, St. Louis, MO, USA) with the most basic formulation (without: glucose, L-glutamine, phenol red, sodium pyruvate, sodium bicarbonate, HEPES) was used to prepare media with pH 7.0 and 6.5. These were defined by the concentration of sodium bicarbonate (Sigma-Aldrich, St. Louis, MO, USA) calculated from the Henderson–Hasselbalch equation below. Acidic media were supplemented with 10% FBS (PAN-Biotech, Aidenbach, Germany) and Pen/Strept (Sigma-Aldrich, St. Louis, MO, USA), and cells were incubated for 48 h. UdSCs were cultured in Pen/Strept-supplemented SFM (Sigma-Aldrich, St. Louis, MO, USA) with acidic pH values with or without RASFs at 1 mg/mL concentration for 48 h.

The Henderson–Hasselbalch equation:

$$pH=pKa+\log\frac{\left[HCO_{3}^{-}\right]}{\left[CO_{2}\right]}$$

Cells were also incubated in SFM hg DMEM (Sigma-Aldrich, St. Louis, MO, USA) with RASFs (1 mg/mL) and recombinant human TNF-α (H8916, Sigma-Aldrich, St. Louis, MO, USA; 10 ng/mL) for 48 h.

### 4.6. ATSCs Treatment Protocol

To evaluate the influence of acidic stress (pH 7.0 and 6.5) on ATSCs, cells were seeded in 96-well plates or 24-well plates. The following day, medium was removed, and cells were washed twice with PBS (Sigma-Aldrich, St. Louis, MO, USA). Control cells were cultured in commercial low-glucose (lg) DMEM (Sigma-Aldrich, St. Louis, MO, USA) supplemented with 10% FBS (PAN-Biotech, Aidenbach, Germany), 2 mM glutamine (Sigma-Aldrich, St. Louis, MO, USA), and Pen/Strept (Sigma-Aldrich, St. Louis, MO, USA) or in commercial lg SFM DMEM (Sigma-Aldrich, St. Louis, MO, USA) supplemented with 2 mM glutamine (Sigma-Aldrich, St. Louis, MO, USA) and Pen/Strept (Sigma-Aldrich, St. Louis, MO, USA) for 48 h. To mimic extracellular acidosis (pH 7.0 and 6.5) for 48 h, DMEM (D5030, Sigma-Aldrich, St. Louis, MO, USA) with the most basic formulation (without: glucose, L-glutamine, phenol red, sodium pyruvate, sodium bicarbonate, HEPES) was used. Acidic media were supplemented with 10% FBS (PAN-Biotech, Aidenbach, Germany), and Pen/Strept (Sigma-Aldrich, St. Louis, MO, USA), and ATSCs were cultured for 48 h. Cells were also kept in Pen/Strept-supplemented SFM (Sigma-Aldrich, St. Louis, MO, USA) with acidic pH values for 48 h.

### 4.7. Morphological Analysis and Cell Area Measurement

The cell morphology of UdSCs and ATSCs was analyzed with a light microscope, Zeiss Axiovert 100 (Carl Zeiss, Jena, Germany) using various magnifications (40×, 100×). For cell area measurement, 25 cells were evaluated from each treatment using ImageJ software v1.48 (NIH, Bethesda, MD, USA).

### 4.8. Proliferation Assay (MTS Test)

Cell proliferation was assessed using tetrazolium-based MTS colorimetric assay. UdSCs were seeded at the density of 0.5 × 10^4^ cells per well in 96-well plates or 0.4 × 10^5^ cells per well in 24-well plates and left to adhere overnight. ATSCs were seeded at the density of 0.5 × 10^4^ cells per well in 96-well plates and also left to adhere overnight. Then, cells were washed twice with PBS and incubated for 48 h as per the protocol described in Section 2.5 and Section 2.6. After 48 h of pre-incubation, an MTS test (Cell Titer 96**^®^** AQueous One Solution Reagent, Promega, Madison, WI, USA) was performed as per the manufacturer’s instructions. Synergy multi-detection plate system HTX (BioTek Instruments Inc., Winooski, VT, USA) was used to measure the absorbance at the wavelength of 490 nm. All MTS tests were performed in quadruplicates.

### 4.9. Cell Viability Test

To assess the impact of extracellular acidosis (pH 7.0 and 6.5) on cell viability/fitness, UdSCs were seeded at the density of 1.245 × 10^6^ cells per T75 flask and ATSCs at 3 × 10^5^ cells per T25 flask. Cells were let to adhere overnight. The following day, standard cultivation medium was aspirated, cells were washed twice with PBS and treated as per protocol described in Section 2.5 and Section 2.6. After 48 h, viability was evaluated. UdSCs as well as ATSCs were trypsinized, and cell suspensions were mixed with trypan blue at 1:1. Then, cells were put into an 8-channel cell counting chamber and analyzed by a CEDEX XS Cell Analyzer (Roche, Basel, Switzerland). The cell viability test was repeated at least three times.

### 4.10. Gene Expression Analysis

UdSCs as well as ATSCs were seeded at the density of 2 × 10^4^ cells in 24-well plates and left to attach overnight. Subsequently, cells were washed twice with PBS and cultured as per the protocol described in Section 2.5 and Section 2.6. Then after 48 h, cells were washed twice with PBS and their pellets were processed for gene expression analysis by reverse transcriptase quantitative PCR (RT-PCR) with TaqMan probes (ThermoFischer Scientific, Waltham, MA, USA) as described previously [21]. Primers (ThermoFischer Scientific, Waltham, MA, USA) for following human genes were used as follows: IL-6, TSG6, ICAM-1, VCAM-1, and PD-L1. Human glyceraldehyde-3-phosphate dehydrogenase (GAPDH) served as an internal control. Relative expression levels of the selected genes were measured by the ∆∆ CT method.

### 4.11. Secretome Analysis

To measure the concentration of selected secreted proteins in conditioned media the MILLIPLEX**^®^** Human Cytokine/Chemokine/Growth Factor Panel A (Merck, Darmstadt, Germany) was applied. Detection of fluorescent signal was performed on Luminex MAGPIX**^®^** Instrument (Luminex, Austin, TX, USA) according to the manufacturer’s instructions. UdSCs were seeded at the density of 2 × 10^4^ cells in 24-well plates and left to adhere overnight. Subsequently, cells were washed twice with PBS and cultured as per the protocol in Section 2.5. Afterwards, cells were washed twice with PBS and kept in SFM hg DMEM for another 48 h to prepare conditioned media. Cell-free supernatants were then collected and analyzed. Data were processed using Belysa^TM^ software v1.2 (Belysa™ Immunoassay Curve Fitting Software, MilliporeSigma (Merck KgAA), St Louis, MO, USA).

### 4.12. Cell Lysis and Western Blotting

UdSCs were seeded at the density of 6 × 10^5^ cells per well in 6-well plates and left to adhere overnight. Then, cells were washed twice with PBS and incubated for 48 h as per the protocol described in Section 2.5. Post-incubation UdSCs were washed with ice-cold PBS and lysed in RIPA Buffer (9806S, Cell Signaling, Danvers, MA, USA) supplemented with Complete Protease Inhibitors (Roche, Basel, Germany) for 15 min on ice. Lysates were then centrifuged at 13,000× *g* at 4 °C for 15 min. Samples containing 30 μg of total proteins were mixed with 4x Laemmli sample buffer (0.01% SDS, 10% glycerole, 30 mM Tris-HCl pH 6.8, 0.005% bromophenol blue, 5% 2-mercaptoethanol), separated by 10% SDS-PAGE followed by immunoblotting and detection with rabbit anti-IκBa (44D4, Cell Signaling, Danvers, MA, USA) antibody diluted at 1:1000 and mouse anti-β-actin (8H10D10, Cell Signaling, Danvers, MA, USA) antibody diluted at 1:1000. HRP-conjugated secondary antibodies (anti-rabbit IgG and anti-mouse IgG) were also purchased from Cell Signaling. The protein bands were visualized with ECL (SuperSignal^TM^, ThermoFischer Scientific, Waltham, MA, USA) and chemiluminescent signals were detected with Li-Cor Odyssey Fc Imaging System (Li-Cor Corporation, Lincoln, NE, USA).

### 4.13. Peripheral Blood Mononuclear Cells (PBMCs) Isolation and Co-Culture with UdSCs

UdSCs were seeded at the density of 4 × 10^4^ cells in 24-well plates and left to attach overnight. Subsequently, cells were washed twice with PBS and cultured as per the protocol described in Section 2.5. Then, after 48 h, the ability of UdSCs to modulate peripheral blood mononuclear cells (PBMCs) was tested.

After obtaining consent, PBMCs were isolated from the peripheral blood of two healthy male donors of ages 35 and 45 using Ficoll-Paque^TM^ PLUS (GE Healthcare, Danderyd, Sweden). Briefly, PBS-diluted blood (1:1) was carefully loaded over the density gradient without mixing. After centrifugation (30 min, 400× *g*), the interface layer of the PBMCs was collected, washed three times with PBS, and counted [39]. The purity and cellular composition of isolated PBMCs were determined by a 7-Color Immunophenotyping Kit (Miltenyi Biotec, Bergisch Gladbach, Germany) according to the manufacturer’s instructions. Subsequently, PBMCs were stained with 5μM carboxyfluorescein succinimidyl ester (CFSE, Sigma-Aldrich, St. Louis, MO, USA) in PBS containing 5% (*v*/*v*) of heat-inactivated FBS (FBS^HI^) for 5 min at room temperature in the dark, according to a previously published protocol [40]. Afterward, CFSE-labeled PBMCs were added to pretreated and PBS-washed UdSCs. To induce PBMCs proliferation, cells were stimulated with phytohaemagglutinin (PHA, Sigma-Aldrich, St. Louis, MO, USA) at a concentration of 10 µg/mL. PBMCs were cultured in the presence or absence of pretreated UdSCs at a UdSC:PBMC ratio of 1:5 for 4 days in RPMI medium (Sigma-Aldrich, St. Louis, MO, USA) supplemented with 10% FBS^HI^ and antibiotics.

### 4.14. Flow Cytometry

After 4 days of co-culture, the CFSE dilution in PBMCs was evaluated by a MACSQuant Analyzer 10 flow cytometer and further analyzed with MACS Quantify software v2.13 (Miltenyi Biotec, Bergisch Gladbach, Germany). Unlabeled and CFSE-labeled PBMCs, with or without PHA (10 µg/mL), in the absence of UdSCs were used as controls. The CFSE-labeled PBMCs without PHA stimulation were used to set the interval gate for proliferating cells determined in percentages. The percentage of events within the ‘PBMCs proliferation’ gate indicates a dilution of CFSE fluorescence, which corresponds to cell division.

### 4.15. Statistical Analysis

Quantitative data were shown as mean ± standard deviation (SD). Values were tested for normality and statistical significance was calculated using one-way ANOVA followed by Bonferroni and Holm post hoc tests for multiple comparisons or two-tailed Student’s *t*-test. * *p* < 0.05; ** *p* < 0.01; *** *p* < 0.001 were considered as statistically significant. All experiments were performed in triplicate or quadruplicate and were repeated at least three times.

## 5. Conclusions

In conclusion, our results suggest that UdSCs can be used as a suitable non-invasive alternative source of not only donor-specific autologous cells but also allogeneic cells for potential cell-based therapies. In the RA scenario, their intra-articular delivery to inflamed joints appears to be a reasonable strategy to mediate the stimulatory effect of the synovial RA microenvironment on the UdSCs’ immunomodulatory activity. However, clarifying the pathways that regulate the regenerative potential of UdSCs in preclinical models remains of critical importance for ameliorating cell-based therapy in RA treatment.

## Figures and Tables

**Table 1:** Demographic and clinical data.

	Age	Sex	Primary Diagnosis	RA-Associated Diagnosis	Systemic Treatment	Local Therapy	RF
RA1	48	Female	Seronegative RA		MTX, Leflunomide, Diclofenac	Depomedrol, Mesocain	-
RA2	59	Female	RA	Gonarthrosis st. II	MTX, Prednisone	Depomedrol, Mesocain, HA	+
RA3	61	Female	RA	Gonarthrosis st. III	Aceclofenac	Depomedrol, Mesocain, HA	+
RA4	82	Male	RA	Gonarthrosis st. III	Naproxen, Aclasta	Depomedrol, Mesocain, HA	+
RA5	24	Female	Seronegative RA		Depo-Medrol	Depomedrol, Mesocain	-
RA6	71	Female	Seronegative RA		MTX, Aceclofenac	Depomedrol, Mesocain, HA	-
RA7	75	Female	RA	Gonarthrosis st. III	Depo-Medrol	Depomedrol	?
RA8	79	Female	RA	Gonarthrosis st. III	Depo-Medrol	Depomedrol, Mesocain	+

RASFs were obtained from 8 RA patients with different stages of disease. HA, hyaluronic acid; MTX, methotrexate; RA, rheumatoid arthritis; RF, rheumatoid factor.

**Table 2:** Cell viability of ATSCs after 48 h pre-treatment in lg DMEM with an acidic pH.

48 h Incubation	Viability
10% FBS lg DMEM	83%
10% FBS lg DMEM pH 7.0	80%
10% FBS lg DMEM pH 6.5	70%
SFM lg DMEM	65%
SFM lg DMEM pH 7.0	64%
SFM lg DMEM pH 6.5	34%

## Data Availability

The data used to support the findings of this study are available from the corresponding author upon reasonable request.
